# Elucidation of Novel Structural Scaffold in Rohu TLR2 and Its Binding Site Analysis with Peptidoglycan, Lipoteichoic Acid and Zymosan Ligands, and Downstream MyD88 Adaptor Protein

**DOI:** 10.1155/2013/185282

**Published:** 2013-07-15

**Authors:** Bikash Ranjan Sahoo, Madhubanti Basu, Banikalyan Swain, Manas Ranjan Dikhit, Pallipuram Jayasankar, Mrinal Samanta

**Affiliations:** ^1^Fish Health Management Division, Central Institute of Freshwater Aquaculture (CIFA), Kausalyaganga, Bhubaneswar, Odisha 751002, India; ^2^Biomedical Informatics Centre, Rajendra Memorial Research Institute of Medical Sciences, Agamkuan, Patna, Bihar 800007, India

## Abstract

Toll-like receptors (TLRs) play key roles in sensing wide array of microbial signatures and induction of innate immunity. TLR2 in fish resembles higher eukaryotes by sensing peptidoglycan (PGN) and lipoteichoic acid (LTA) of bacterial cell wall and zymosan of yeasts. However, in fish TLR2, no study yet describes the ligand binding motifs in the leucine rich repeat regions (LRRs) of the extracellular domain (ECD) and important amino acids in TLR2-TIR (toll/interleukin-1 receptor) domain that could be engaged in transmitting downstream signaling. We predicted these in a commercially important freshwater fish species rohu (*Labeo rohita*) by constructing 3D models of TLR2-ECD, TLR2-TIR, and MyD88-TIR by comparative modeling followed by 40 ns (nanosecond) molecular dynamics simulation (MDS) for TLR2-ECD and 20 ns MDS for TLR2-TIR and MyD88-TIR. Protein (TLR2-ECD)–ligands (PGN, LTA, and zymosan) docking in rohu by AutoDock4.0, FlexX2.1, and GOLD4.1 anticipated LRR16–19, LRR12–14, and LRR20-CT as the most important ligand binding motifs. Protein (TLR2-TIR)—protein (MyD88-TIR) interaction by HADDOCK and ZDOCK predicted BB loop, **α**B-helix, **α**C-helix, and CD loop in TLR2-TIR and BB loop, **α**B-helix, and CD loop in MyD88-TIR as the critical binding domains. This study provides ligands recognition and downstream signaling.

## 1. Introduction

The innate immune response elicited by a variety of pattern recognition receptors (PRRs) is an immediate nonspecific and first line of defense of the host against invading various pathogens [1]. Toll-like receptors (TLRs) are a component of PRR and play a critical role by sensing organisms ranging from protozoa to bacteria and were involved in many infectious diseases [2]. They recognize a wide array of microbe associated molecular patterns (MAPMs) and activate downstream signaling to induce innate immunity [3]. The number of TLRs varied in different organisms and among these most TLRs are located on cell surface except for TLR3, TLR7, TLR8, and TLR9 [4, 5].

 Toll-like receptor 2 (TLR2) was shown to be the principal mediator of macrophages activation. It functions as homodimer [6] or heterodimer with TLR1 or TLR6 [7] to recognize diverse bacterial products [8] and activation of MyD88-dependent signaling pathway. In this pathway, the TIR domain of MyD88 interacts with the TIR domain of TLR [9] and transmits downstream signals to induce innate immune genes expression. 

PGN is a highly complex structural component and an important derivative of both Gram-positive and Gram-negative bacterial cell wall, and is the target of the innate immune system [10]. It is composed of alternating *β*-(1–4)-linked N-acetyl-glucosamine and N-acetyl-muramic acid residues cross-linked by peptide bridges [11]. It is recognized by various families of PRRs, including TLRs, nucleotide-binding oligomerization domain-containing proteins (NLRs), and peptidoglycan recognition proteins (PGRPs) [12]. In monocytes and macrophages, PGN binds to extracellular domain of TLR2 and activates signaling to induce inflammatory cytokines [13–15]. Structurally, PGN of most Gram-positive bacteria contains lysine at third position, and in Gram-negative and most rod-shaped Gram-positive bacteria lysine is replaced by DAP [16]. Nascent PGN of bacterial cell wall is poorly recognized by TLR2. However, after its autolysin the remodeled PGN binds TLR2 with high affinity [16]. LTA is an amphiphilic, negatively charged glycolipid [17] component of Gram-positive bacteria cell wall. TLR2 binds LTA and activates signaling cascade to induce TNF-*α*, IL-6, and IL-8 gene expression [18–20]. Zymosan is the cell wall derivative of *Saccharomyces cerevisiae*. It comprised mainly polysaccharides, of which *β*-glucan and mannan are the major constituents. It was widely used as a model to study fungus-mediated inflammation, phagocytosis, and the production of inflammatory cytokines and chemokines [21, 22]. TLR2 recognizes it directly or in coaction with CD14 and TLR6 [23, 24] to induce TNF-*α* gene expression [25]. TLR2 is the major pathway of proinflammatory signaling by zymosan interaction and is needed for the development of specific immune responses against pathogens [26]. 

Various studies on TLR2 have also been reported in zebrafish [27, 28], Japanese flounder [29], puffer fish [30], channel catfish [31], and in orange-spotted grouper [32, 33]. In European common carp, inductive over expression of TLR2 in macrophages was observed in response to PGN and LTA [34]. In the Indian major carps, modulation of TLR2 expression was reported in PGN, zymosan, and LTA treatment [35, 36].

India is the major supplier of fish in the world and ranks 3rd in freshwater fish production (FAO). Among various freshwater fishes, rohu (*Labeo rohita*) is the most commercially important and highly favored fish in the Indian subcontinent. TLR2 was characterized in rohu and the ligands that stimulate TLR2 signaling were also reported [35]. However, no studies have reported yet describing the structural characteristics of TLR2 in rohu and their key domains that binds to the specific ligands to stimulate cytokine expression. Furthermore, the key amino acids in the TLR2-TIR domains that interact with adapter molecule MyD88 to induce down-stream signaling were still unclear across the species.

To elucidate the structural scaffold in rohu TLR2, we report the 3D-model of extracellular domain of rohu TLR2 along with its key domains that are predicted to be involved in recognizing PGN, LTA and zymosan, and the critical region of interaction between TIR domains of TLR2 and MyD88. This is the first report across the fish species. 

## 2. Materials and Methods

### 2.1. Domain Identification

 Rohu TLR2 protein (GenBank ID: ADQ74644) with N-terminal extracellular domain (ECD), transmembrane domain, and C-terminal cytoplasmic TIR domain [35] was subjected to SignalP 4.1 server (http://www.cbs.dtu.dk/services/SignalP/) and NetNGlyc1.0 server (http://www.cbs.dtu.dk/services/NetNGlyc/) to predict the signal peptide and N-glycosylation sites respectively. The TIR domain in common carp MyD88 (GenBank ID: ADQ08685) was predicted by SMART (http://smart.embl-heidelberg.de/) and CD-search (http://www.ncbi.nlm.nih.gov/Structure/cdd/wrpsb.cgi) domain finding programs and was verified with published report of MyD88-TIR domains in zebrafish (Q5XJ85) and puffer fish (A8QMS7) in UniProt database (http://www.uniprot.org/).

### 2.2. Sequence Alignment, Template Identification, and Comparative Modeling of ECD and TIR Domain in Rohu TLR2, and MyD88-TIR Domain in Common Carp

Amino acid sequence of rohu TLR2 was aligned by MegAlign [37] in DNASTAR-Lasergene program with the amino acid sequences of TLR2 in other species derived from UniProtKB database. The TIR domains of rohu TLR2 and common carp MyD88 were aligned by MegAlign with amino acid sequences of TIR domains in other species deduced from UniProtKB database. The secondary structures of TLR2- and MyD88-TIR domains were predicted by PSIPRED program (http://bioinf.cs.ucl.ac.uk/psipred/). 

Template search for TLR2-ECD (561 aa), TLR2-TIR (146 aa), and MyD88-TIR (137 aa) domains in PDB database identified mouse TLR1-TLR2 heterodimer (PDB ID: 2Z81), TIR domain of human TLR2 (PDB ID: 1O77), and TIR domain of human MyD88 (PDB ID: 2Z5V) as the best homologous structures with top identity score. To ascertain the sensitivity and accuracy of the selected templates, FUGUE [38] program was used to perform sequence-structure comparison between the target and the template and was represented by JOY annotation program [39]. For each three domains (TLR2-ECD, TLR2-TIR, and MyD88-TIR) a set of twenty 3D models were generated by Modeller9v10 program [40]. Among these 20 models (for each domain), the model with lowest discrete optimized protein energy (DOPE) score was considered for further studies. The lowest DOPE models of TLR2-ECD, TLR2-TIR, and MyD88-TIR were subjected for loop modeling and refinement in Accelrys DS 2.5 (San Diego, Accelrys) under CHARMM force field. The long BB loops and DD loops in TLR2-TIR and MyD88-TIR models after loop refinement were notably analyzed, and changes were marked by superimposing them with their respective templates. The refined models were subjected to energy minimization by DS 2.5.

### 2.3. Molecular Dynamics Simulation

Molecular dynamics (MD) simulations were carried out for the modeled systems using the GROMACS 4.5.5 program [41]. Homology models were set for MDS under GROMOS54a7 force field. The 3D models were placed in a cubic box maintaining a distance of 10 Å for TLR2-ECD, 9 Å  for TLR2-TIR, and 9 Å for MyD88-TIR between the box edges and the protein surface. The systems were solvated in simple point charge (SPC) models and were neutralized by adding counter ions. In order to remove spurious contacts energy minimization of the solvated systems was done using the steepest descent integrator. The bond lengths and geometry of water molecules were constrained. All of the three restrained models were subjected to position-restrained MD under NPT conditions for 1 ns (nanosecond). Finally, 40 ns production MD run was carried out for TLR2-ECD and 20 ns for TLR2-TIR and MyD88-TIR models using particle mesh Ewald (PME) electrostatics method under NPT conditions. Snapshots of the trajectory were taken in every 0.5 picoseconds. GROMACS and VMD 1.9.1 (http://www.ks.uiuc.edu/Research/vmd/) routines were utilized to check trajectories and the quality of the simulations. The graphs of trajectory analysis were created using Xmgr 4.1.2 (http://plasma-gate.weizmann.ac.il/Xmgr/). 

### 2.4. Model Validation

The final snapshot obtained at the end of each MDS was considered to represent the structures of the TLR2-ECD, TLR2-TIR, and MyD88-TIR models. These simulated models were set for validation by SAVES (http://nihserver.mbi.ucla.edu/SAVES/), WHAT IF [42], MolProbity [43], ProQ [44], ModFOLD [45], and MetaMQAP [46] servers. The simulated models were superimposed with their respective templates to examine the deflections by PyMOL (http://www.pymol.org/). Cross-check validation was carried out using model as template and the primary amino acids of the respective template as target. 

### 2.5. Molecular Docking of PGN, LTA, and Zymosan with Rohu TLR2-ECD

Three different 2D structures of PGN [16], that is, (i) MurNac-L-Ala-*i*-D-Glu-L-Lys (PGN-I), (ii) MurNac-L-Ala-*i*-D-Glu-L-Lys-D-Ala-Gly (PGN-II), and (iii) MurNac-L-Ala-*i*-D-Glu-L-DAP-D-Ala (PGN-DAP), were generated by Chemsketch (http://www.acdlabs.com/resources/freeware/chemsketch/). The 2D structure of zymosan (CID: 11375554) was obtained from the NCBI PubChem database (http://pubchem.ncbi.nlm.nih.gov/) and LTA was from KEGG (KEGG: C06042) ligand database (http://www.genome.jp/ligand/). The 3D structures of all these compounds were generated using PRODRG2 server (http://davapc1.bioch.dundee.ac.uk/prodrg/) subjecting to chirality, full charges with energy minimization. The generated 3D structures were subjected to DS 2.5 for ligand minimization. The probable ligand binding pockets in TLR2-ECD were predicted by metaPocket finder [47] and Q-site finder [48]. The LTA binding site in mouse TLR2 (PDB ID: 3A7B) was also considered for docking. Molecular docking was carried out using AutoDock 4.0 [49], FlexX 2.1 [50], and GOLD 4.1 [51] following previously described methods [52] with receptor and ligand flexibility. In this, the important neighbouring residues at the predicted binding sites were set to flexible that covered all the active site residues and allowed for the flexible rotation of the ligand. Docking of previously reported PGN binding sites in other species [53] was also carried out. In AutoDock, the lowest-energy solution of the ligand all-atom RMSD cluster was taken to calculate the binding energy. The predicted interacting residues obtained by AutoDock were matched with the predicted binding pocket amino acids of metaPocket finder and Q-site finder, and these binding pockets were referred for docking in FlexX and GOLD. The docking poses with H-bond forming amino acids were graphically represented by PyMOL and DS 2.5. 

### 2.6. Protein-Protein Interaction

Rohu and common carp (*Cyprinus carpio*) belong to the Cyprinidae family and are very closely related. Till date, rohu MyD88 gene has not been cloned. Therefore, to understand the TLR2 and MyD88 interaction, we considered common carp MyD88 (GenBank ID: ADQ08685). The interface residues for TLR2-TIR and MyD88-TIR domains were predicted with reference to their template proteins structural and functional properties. Protein-Protein Interaction Site Predictor (cons-PPISP) (http://pipe.scs.fsu.edu/ppisp.html), InterProSurf (http://curie.utmb.edu/prosurf.html), and PatchDock server (http://bioinfo3d.cs.tau.ac.il/PatchDock/) were used to find the interacting residues in TLR2-TIR and MyD88-TIR. Docking was performed using HADDOCK [54] and ZDOCK [55] web servers. Intermolecular contacts were analyzed with DIMPLOT, a part of LIGPLOT software package (http://www.ebi.ac.uk/thornton-srv/software/LigPlus/) using default parameters.

### 2.7. Structural Refinement and Stability Evaluation of Complexes

The best protein-ligand complexes obtained from docking studies of PGN, LTA, and zymosan with TLR2-ECD were subjected to MDS using the previously defined parameters in GROMACS. To gain insight into the structural stability of the protein-ligand and protein-protein complexes, MD simulations were performed for PGN-I-TLR2-ECD, PGN-II-TLR2-ECD, PGN-DAP-TLR2-ECD, LTA-TLR2-ECD, zymosan-TLR2-ECD, and TLR2-TIR-MyD88-TIR complex for different time periods of MDS. A production MD run for 10 ns was carried out for TLR2-ECD ligand complexes and protein-protein complex. The existence of H-bonds in the complex in different periods of MDS was analyzed.

### 2.8. In Silico Site-Directed Mutagenesis

To identify the key amino acids among interacting amino acid residues in TLR2-ECD, TLR2-TIR, and MyD88-TIR domains, site-directed mutagenesis was carried out in DS 2.5 under build mutant protocol. Redocking was performed to calculate the fitness score in GOLD after mutation, and docking scores were cross-checked with previous fitness scores. Protein-protein interaction hot spots were predicted after mutagenesis by HADDOCK.

## 3. Results and Discussion

### 3.1. Domain Analysis

**Figure 1:**
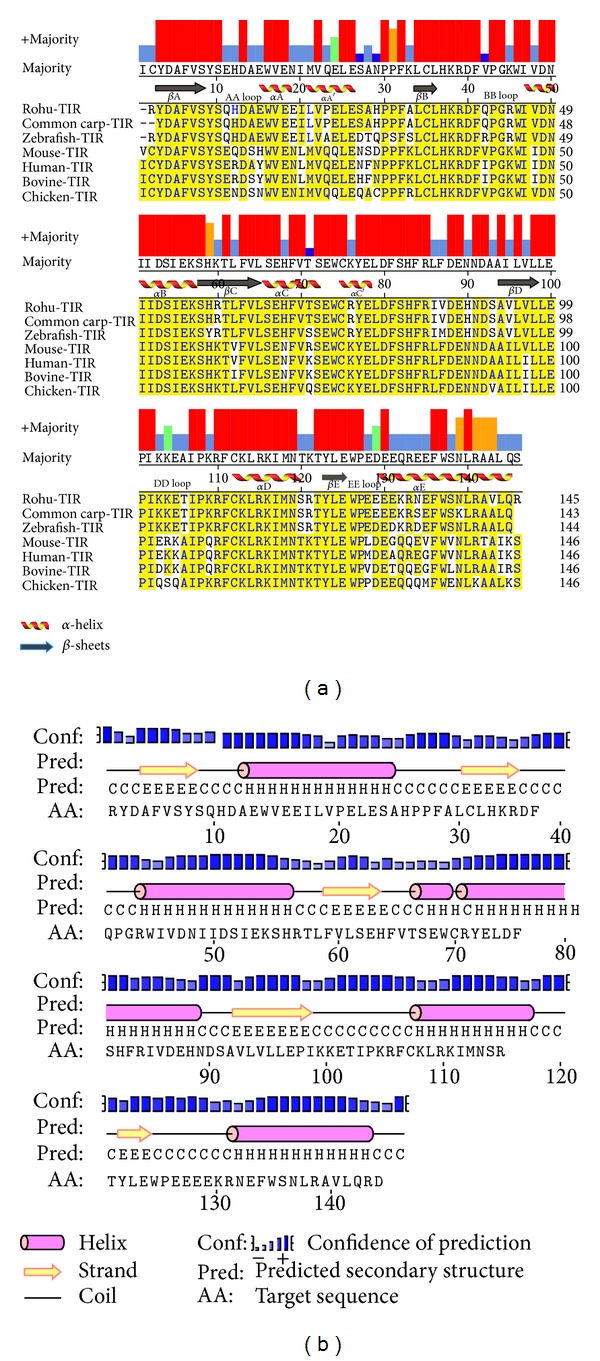
Multiple sequence alignment and secondary structure prediction of TLR2-TIR domain. (a) Multiple sequence alignment of TLR2-TIR domain of rohu with others by MegAlign program. Conserved residues were shown in yellow. Consensus residues are shown in the majority axis. (b) Secondary structure representation of TLR2-TIR domain by PSIPRED. Helices denoted as “H,” beta strands as “E,” and loops as “C.”

**Figure 2:**
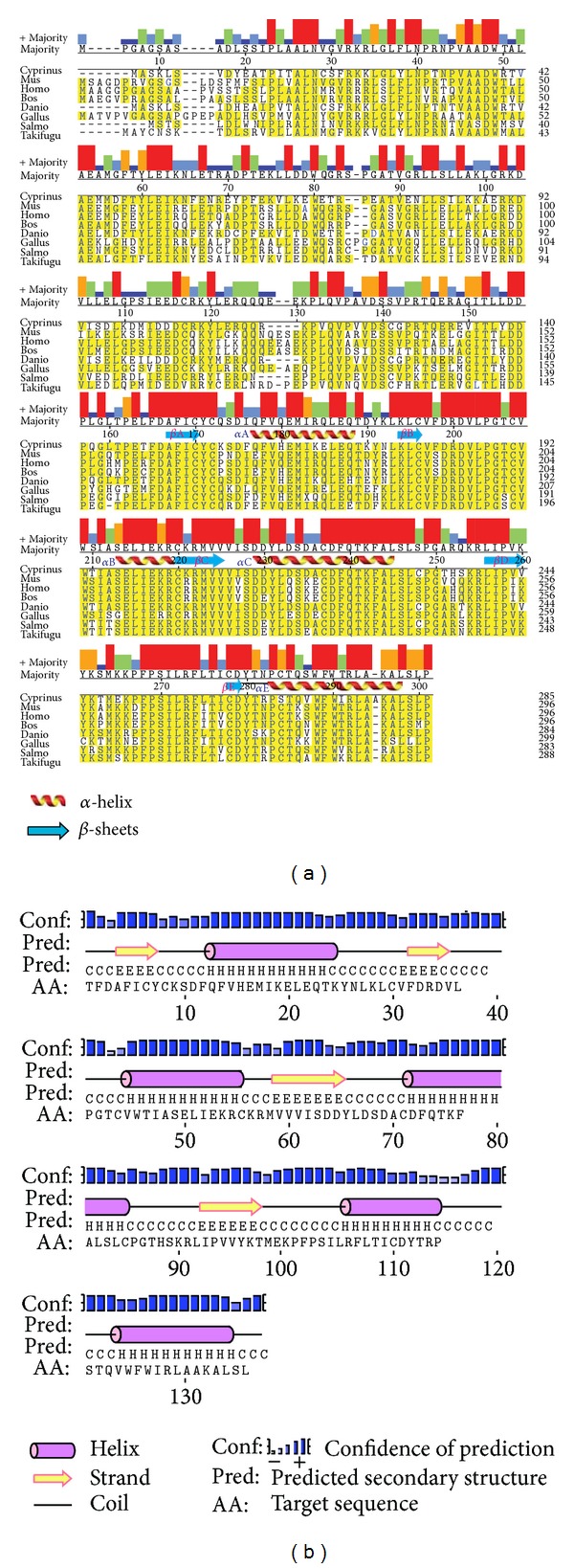
Multiple sequence alignment and secondary structure prediction of MyD88-TIR domain. (a) Multiple sequence alignment of MyD88-TIR domain of common carp with others by MegAlign program. Conserved residues were shown in yellow. Consensus residues are shown in the majority axis. (b) Secondary structure representation of MyD88-TIR domain by PSIPRED. Helices denoted as “H,” beta strands as “E,” and loops as “C.”

The full length TLR2 protein is constituted of 792 amino acids including a signal peptide of 30 amino acids (1–30 aa). The mature TLR2 protein ECD, trans-membrane (TM) and TIR domain constituted of 34–590, 595–612, and 645–790 amino acids respectively. The alignment of TLR2 amino acids with other species revealed their good conservation across the species (See Figure S1 in supplementary material available online at doi: http://dx.doi.org/10.1155/2013/185282). Among them, rohu TLR2 showed highest identity with common carp (88.1%) and lowest identity with mouse (40.5%). N-glycosylation site prediction server predicted 10 glycosylation sites, out of which 9 were in the ECD and 1 in the TIR domain. Among these 10 N-glycosylation sites, 8 were potential with a value greater than threshold value (0.5) and the remaining two were below the threshold. Single N-glycosylation site was present each at LRR1, 3, 8, 14, 16, 18, 21, and TIR domain, and the remaining two were at LRR6. The multiple sequence alignment of TLR2-TIR with other species (Figure 1(a)) and secondary structure analysis (Figure 1(b)) showed well-conserved *α*-helices, *β*-sheets, and biologically most important BB and DD loops [56]. In MyD88-TIR domain the multiple sequence alignment and secondary structure prediction (Figures 2(a) and 2(b)) also revealed a good conservation among the phylogenetically divergent species.

### 3.2. Structural Analysis of TLR2-ECD, TLR2-TIR, and MyD88-TIR Domains

The BLAST search analysis showed that the ligand recognizing LRR regions in TLR2-ECD shared the close structural relationship with mouse TLR1-TLR2 heterodimer (PDB ID: 2Z81) having 35% and 52% sequence identities and similarities, respectively. The TLR2-TIR and MyD88-TIR domains shared 71% and 78% sequence identities with their respective templates (PDB ID: 1O77 and 2Z5V). The sequence-structure alignment by FUGUE revealed a good conservation of secondary structures (*α*-helices and *β*-sheets) between the target and template. The identity scores between the LRR regions of TLR2-ECD and 2Z81 were presented in Table 1. The sequence identities between the important biological regions as reported in human and mouse TIR domains with their respective templates were given in Tables 2 and 3. The structure-structure alignment between the lowest DOPE score models and their respective templates showed good structural conservation across the domains. The TLR2-ECD took a horseshoe shape with 23 LRR domains including LRRNT and LRRCT. Most of the LRR domains consisted of *β*-strands connected by long loop and some with *α*-helices. The *β*-strands faced towards the concave surface in TLR2-ECD model, and the *α*-helices were present at the convex surface. There were five *α*-helices and five *β*-sheets in TLR2-TIR domain and four *α*-helices and five *β*-sheets in MyD88-TIR domain. 

### 3.3. Molecular Dynamics of Homology Models

**Figure 3:**
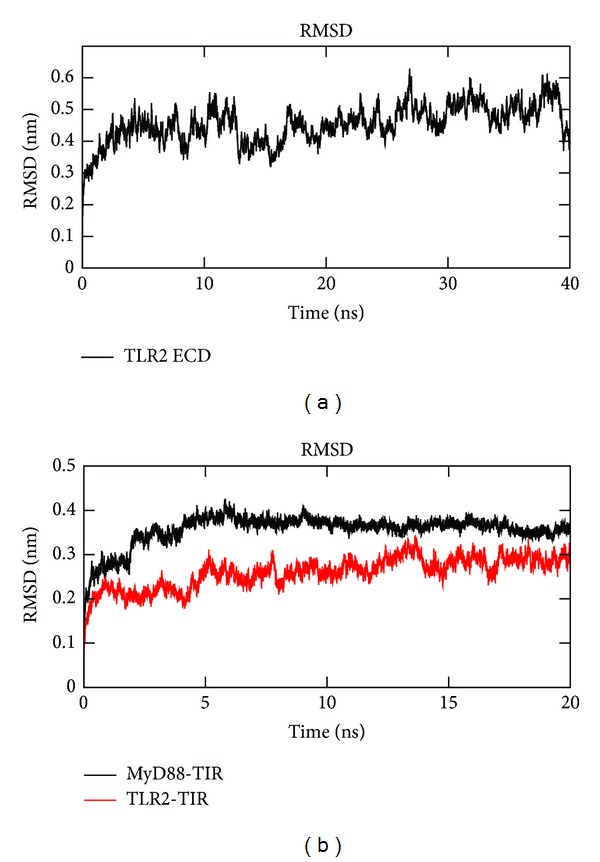
Root mean square deviation (RMSD) analysis. RMSD of (a) TLR2-ECD up to 40 ns and (b) MyD88-TIR and TLR2-TIR domains up to 20 ns MD simulation.

**Figure 4:**
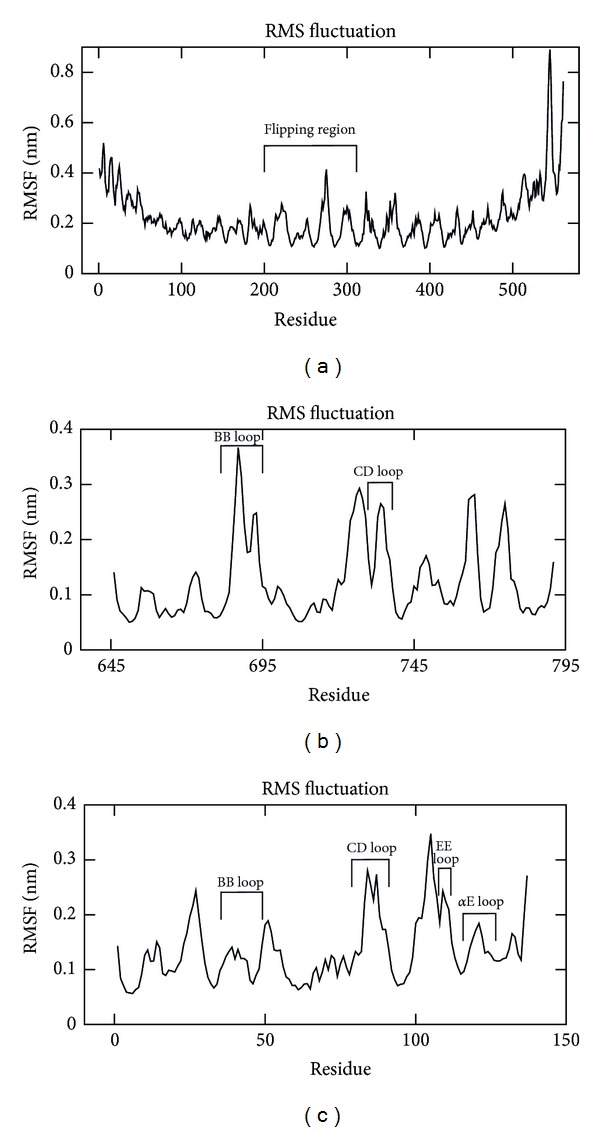
Root mean square fluctuation (RMSF) analysis for homology models. RMSF per residue over the dynamics was shown in graph. (a) TLR2-ECD; (b) TLR2-TIR; (c) MyD88-TIR.

The stability and MD properties were observed up to 40 ns for TLR2-ECD and up to 20 ns for TLR2-TIR and MyD88-TIR domains, and the RMSD values over time were shown in Figure 3. The MD analysis in TLR2-ECD showed that the RMSD trajectory rose from the beginning to 12 ns with an average RMSD of 4.23 Å. It attained an approximately stable plateau with an average RMSD of 4.673 Å till the end of simulation. The RMSD trajectories of TLR2-TIR and MyD88-TIR were observed to be stable after 5 ns with an average RMSD of 2.74 Å and 3.68 Å, respectively. The RMSF of C*α* atoms of each amino acid in TLR2-ECD identified LRR7-11 as the most flipped region (Figure 4(a)). These regions are constituted of six *β*-sheets, one *α*-helix, and long loops. In higher vertebrates, this region was reported as lipopeptide binding region [57]. The flexible long loops (BB and DD loops) in TLR2-TIR and MyD88-TIR domains showed major fluctuations in the RMSF graph and were expected to be engaged in protein-protein interaction (Figures 4(b) and 4(c)). Secondary structure analysis from the trajectory in TLR2-ECD showed little variation of *α*-helices and *β*-sheets. However, the coil regions much varied with respect to simulation period. In TLR2-TIR, no major secondary structural changes were observed during MDS.

### 3.4. Validation of Homology Models

The PROCHECK analysis at SAVES of three models (TLR2-ECD, TLR2-TIR, and MyD88-TIR) showed that the phi-psi angles of most of the residues were in the allowed regions of Ramachandran plot (Figure S2). The SAVES results (Table 4(a)) of all models were within the cut-off range suggesting the reliability of our proposed models. The protein stereochemical quality analysis by ProQ, ModFOLD, and MetaMQAP servers showed an acceptable score of all models (Table 4(b)). The average coarse packing quality, planarity, and the collision with symmetry axis, bond lengths, and bond angles obtained by the WHAT IF server of all models revealed the satisfactory acceptance of the models. The results of MolProbity server of all models were also within the range. The RMSD value for all atoms and C*α* atoms by superimposing target models with their respective templates showed very low deviation along the significant biological domains. The low deviation between the target-template structures suggested the acceptance of the proposed models. The cross-check validation report indicated acceptability between the experimental structures (PDB ID: 2Z81, 1O77, and 2Z5V) and their models. The RMSD values calculated by PyMOL superimposition program for C*α* atoms between the PDB coordinates and the lowest DOPE score models of 2Z81, 1O77, and 2Z5V generated by Modeller were 1.13 Å, 0.518 Å, and 0.705 Å, respectively. The comparison of Ramachandran plot analysis of the homology models of 2Z81, 1O77, and 2Z5V showed similar results for both experimental and hypothetical models (Table S1). The cross-check validation fortified the acceptance of TLR2-ECD, TLR2-TIR, and MyD88-TIR models.

### 3.5. Binding Site Analysis of PGN, LTA, and Zymosan**  **with TLR2-ECD

**Figure 5:**
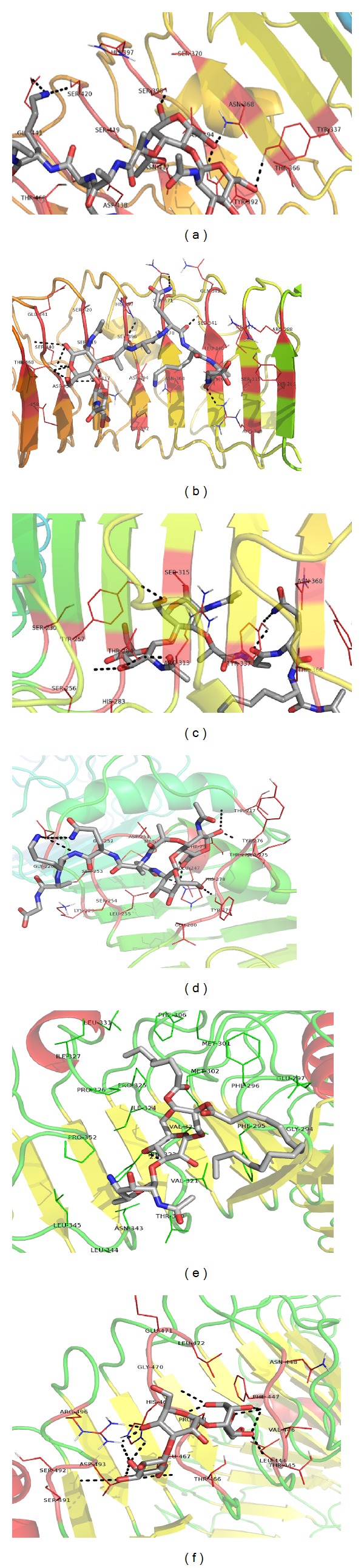
Illustration of the interaction of PGN, LTA, and zymosan with the modeled 3D structure of rohu TLR2-ECD by AutoDock 4.0 program. At B5 region, interaction of PGN-I with TLR2-ECD (a) and PGN-DAP with TLR2-ECD (b); at B6 region, interaction of PGN-I with TLR2-ECD (c) and PGN-DAP with TLR2-ECD (d); interaction of LTA with TLR2-ECD (e) and zymosan with TLR2-ECD (f). The TLR2-ECD was shown in ribbon and ligands (PGN, LTA, and zymosan) were shown in solid form. Amino acid number depicted in the figure was shown as per the matured protein (after removal of the signal peptide).

For docking analysis, the predicted top seven (B1 to B7) probable ligand binding pockets in TLR2-ECD (close to the N-glycosylation sites) were considered (Table S2) including previously reported LTA binding site in mouse TLR2 [58]. Interaction of PGN with TLR2-ECD in AutoDock revealed PGN binding sites at B5, and it was in agreement with the previous observation [53]. Both PGN-I and PGN-DAP showed good interactions at B5 regions (Figures 5(a) and 5(b)). The lists of interacting amino acids were presented in Table 5(a). Docking at B6 site also presented good binding score for PGN ligands (Figures 5(c) and 5(d)). AutoDock identified B_mouse_ and B7 with highest binding affinities for LTA and zymosan, respectively (Figures 5(e) and 5(f)).

In FlexX docking, the binding sites B3 and B4 revealed high positive FlexX score for all ligands and, hence, excluded from further studies. The B5 site (LRR16–19) resulted in good FlexX score −18.85, −12.80, and −15.47 for PGN-I, PGN-II, and PGN-DAP, respectively. Docking at B6 site (LRR8-10) also resulted a satisfactory FlexX score for PGN-II (−13.23) and comparatively a low FlexX score was predicted for PGN-I and PGN-DAP. The rest of the binding pockets were found to be irrelevant for PGN interaction with very little positive and negative FlexX score. The interaction of LTA with TLR2-ECD with highest FlexX score (−6.92) was obtained at B_mouse_ region (LRR12–14). Interaction of LTA at other binding sites yielded irrelevant FlexX score. Zymosan interacted with TLR2-ECD at B7 (LRR20-CT) region effectively with a FlexX score of (−13.81), and other binding sites were found to be very less interactive. The FlexX scores at different binding regions were presented in Table 5(b). 

**Figure 6:**
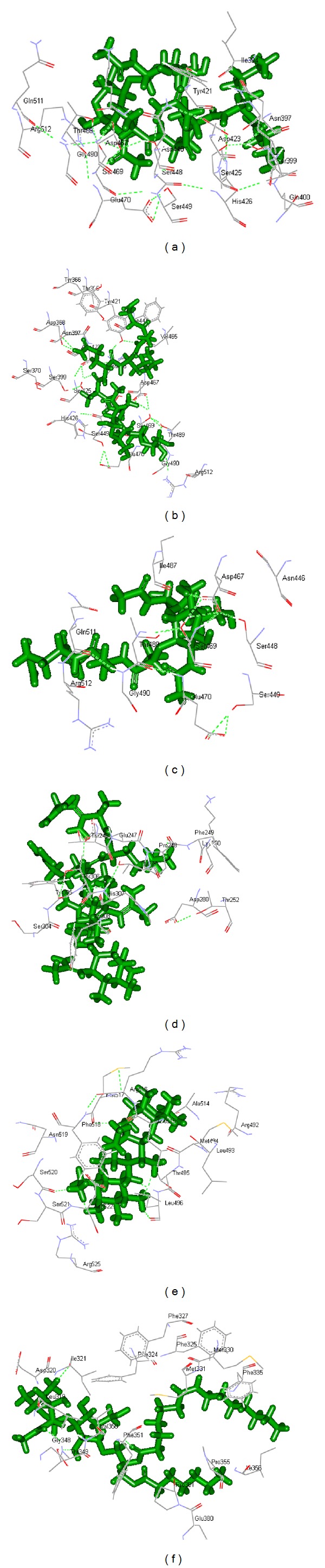
Illustration of the interaction of PGN, LTA, and zymosan with the modeled 3D structure of rohu TLR2-ECD by GOLD 4.1 program. Interaction of (a) PGN-I and TLR2-ECD at LRR16-19; (b) PGN-II and TLR2-ECD at LRR16-19; (c) PGN-DAP and TLR2-ECD at LRR16-19; (d) PGN-II and TLR2-ECD at LRR8-10; (e) zymosan and TLR2-ECD at LRR20-CT; (f) LTA and TLR2-ECD at LRR12-14. Rohu TLR2-ECD model was shown as line and ligands (PGN, LTA, and zymosan) are shown as stick.

The GOLD scores for PGN-I, PGN-II, and PGN-DAP at B5 and B6 sites, for LTA at B_mouse_ and for zymosan at B7 site were given in Table 5(c). The GOLD fitness score was found to be highest in the proposed binding sites of FlexX program in comparison to other binding sites (Figures 6(a)–6(f)). The GOLD docking score of PGN-I, PGN-II, and PGN-DAP was predicted to be less at B6 site as compared to B5 site (Figures 6(a)–6(c)). Interaction of PGN-II at B6 site also showed a good GOLD fitness score (Figure 6(d)). Docking of zymosan (Figure 6(e)) and LTA (Figure 6(f)) in GOLD also ensured B7 and B_mouse_ as the potential binding sites. 

### 3.6. TLR2-TIR and MyD88-TIR Interaction

**Figure 7:**
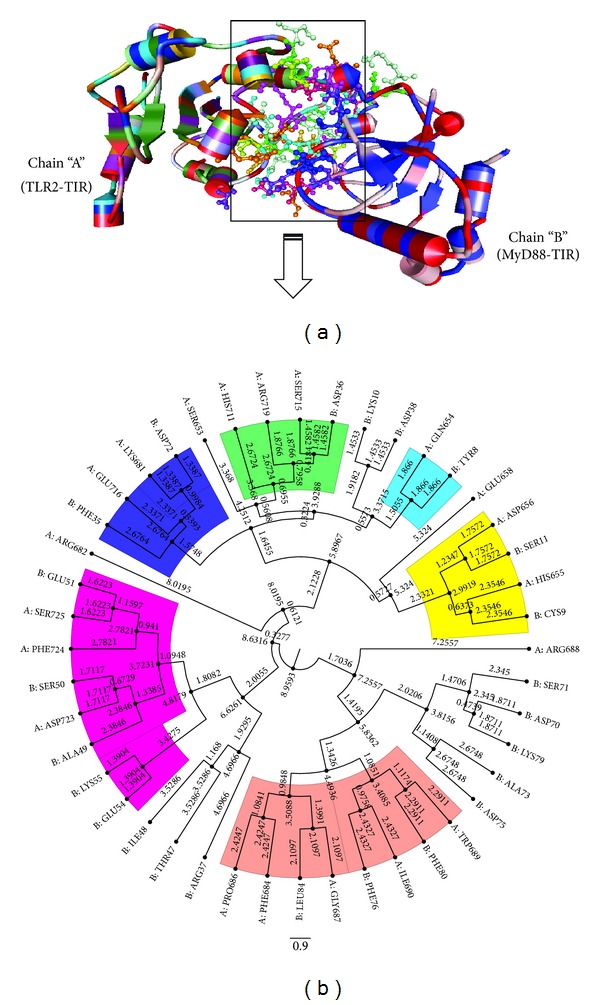
Interaction of TLR2-TIR and MyD88-TIR in Discovery Studio 2.5. (a) TLR2-TIR model is labeled as chain “A” and MyD88-TIR model is labeled as chain “B.” Interface residues are shown inside a rectangle box in ball and stick representation. (b) Clustering of interface residues between TLR2-TIR and MyD88-TIR domain in tree format. Residues of TLR2-TIR are marked as chain “A” and MyD88-TIR as chain “B.” Strongly interacting residues are highlighted with different colors.

The MyD88 functions as an adaptor molecule that transmits signal to downstream molecules from ligand activated TLRs by interacting with the TIR domains. The predicted interface residues in cons-PPISP, InterProSurf, and PatchDock were observed to be present in the most flexible regions of TLR2-TIR and MyD88-TIR domains (Table S3). Majority of the interacting amino acids were distributed in BB loop, *α*B, *α*C, and CD loop in TLR2-TIR and BB loop, *α*B helix, and CD loop in MyD88-TIR (Table S3). The best cluster obtained in HADDOCK showed a very low RMSD (1.2 ± 0.7 Å) and intermolecular energy (−722.9 kcal mol-1) with a buried surface area of 2029.2 Å^2^. The binding orientation and amino acid interactions generated by DS 2.5 were presented in Figure 7(a). The phylogenies of interacted amino acids identified the strongly bonded residues and were highlighted in Figure 7(b). DIMPLOT analysis of the complex showed that BB loop, *α*C, and *α*C′ helix residues of TLR2-TIR domain were mostly interacted with AA, BB loops and *α*B, *α*C helices of MyD88-TIR domain (Figure S3(a)). The protein-protein complex (Rank-1) in ZDOCK yielded approximately same interacting amino acids residues (as HADDOCK) for TLR2-TIR and MyD88-TIR domains (Figure S3(b)). Amino acid residues in TLR2-TIR and MyD88-TIR domain involved in hydrogen bonding and hydrophobic interactions were presented in Table 6. 

### 3.7. Stability Analysis of Protein Complexes by MDS

**Figure 8:**
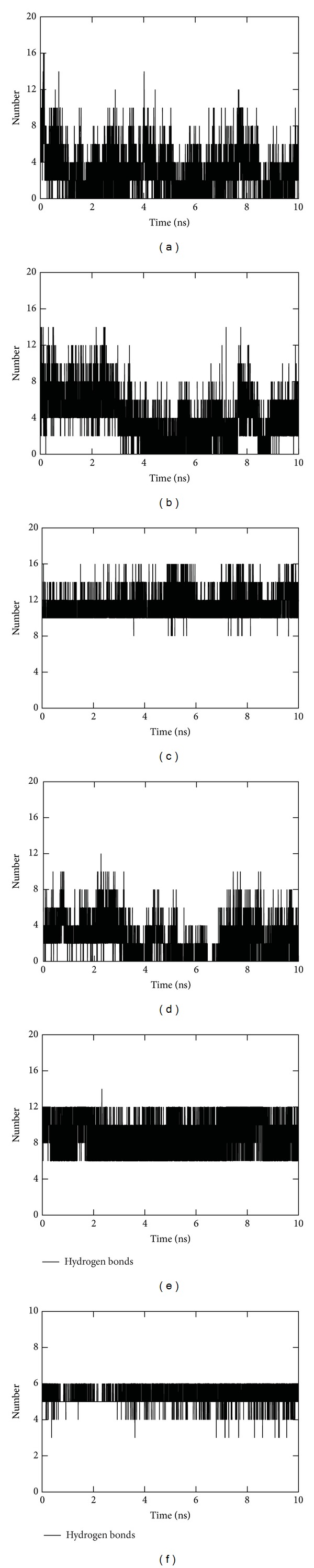
Hydrogen bond analysis of protein-ligand complexes. H-bond analysis of (a) PGN-I and TLR2-ECD complex, (b) PGN-II and TLR2-ECD complex, (c) PGN-DAP and TLR2-ECD complex, (d) PGN-II and TLR2-ECD complex at LRR8-10 region (B6), (e) LTA and TLR2-ECD complex at LRR20-CT, (f) and zymosan and TLR2-ECD complex at LRR12-14. *X*-axis represents time period of simulation and *Y*-axis represents number of hydrogen bonds.

Previously it was reported that binding site B5 (LRR16–19) (corresponding to human) had the highest affinity for PGN recognition, and binding site B6 (LRR8–10) had a low potential for PGN recognition [53]. In this study, docking scores in B6 for PGN were comparatively lower than B5 region. However, the number of N-glycosylation sites closer to B6 was higher. This data may suggest the possibility of PGN interaction as it is comprised of N-acetylglucosamine. The H-bond analysis showed more number of H-bonds in B5 region (avg. 8 numbers) than B6 region (avg. 5 numbers) with highest binding affinity (Figures 8(a)–8(c)). The existence of H-bonds fluctuated at B6 region during different time periods of MD simulation (Figure 8(d)). Thus, both the docking analysis and MDS suggested B5 region (LRR16–19) as the possible PGN recognition site in rohu TLR2. H-bond analysis for LTA and zymosan at B_mouse_ (LRR12–14) and B7 (LRR20-CT) region depicted a stable orientation with conserved number of H-bonds throughout the simulation (Figures 8(e) and 8(f)). The DIMPLOT analysis of TLR2-TIR and MyD88-TIR complex generated after 10 ns of MDS showed that the amino acid residues that were involved in protein-protein interaction were retained after the MDS (Figure S4). This suggested that the predicted interacting loops and helices in TLR2-TIR and MyD88-TIR domains were significant for protein-protein interaction.

### 3.8. Validation of Binding Domains by Site-Directed Mutagenesis

Alanine scanning of B5 regions of PGN binding domains showed loss of interaction due to absence of donors or acceptors in the active sites. But no single mutation of alanine for the interacting residues at this region showed a complete loss of docking. Proline and aspartic acid scanning of B5 region also ensued very low fitness score (9.6 and 12.8) in GOLD. Analysis of B5 region by mutating residues in pair, triplet, and quadruple combinations at a time indicated the viable importance of Asp394, Ser396, Asn397, and Ser399 as the fitness score attained a very minimum value in comparison to other GOLD runs. 

Mutagenesis study at B7 residues in TLR2-ECD that formed H-bond with zymosan revealed a good fitness score for alanine scanning. However, proline scanning of all residues revealed loss of zymosan interaction with TLR2-ECD. Single proline mutation and acidic to basic mutation of different interacting residues followed by individual GOLD runs explored the importance of Ser520 and Asp522. A single mutation of Ser520-Pro520 and Asp522-Gln522 resulted in complete loss of zymosan interaction. Mutation of other residues altered the GOLD fitness score; however, in none of the cases docking loss was ensued. Alanine and proline scanning of LTA binding site resulted in low docking score emphasizing the role of key residues Asp320 and Phe324 in LTA recognition. 

## 4. Conclusion

The proposed 3D model of rohu TLR2 describes the protein features and its important domains. It also accentuates the importance of predicting key amino acids and LRR regions that are responsible for the specific ligand interaction and TLR2 signaling in fish and depicts a residue-detailed structural theoretical model. In the absence of crystal structure of TLR2 in any fish, this study provides structural insight into the TLR2 domains architecture. In rohu fish, the peptides at LRR16–19 (at B5), LRR12-14 (at B_mouse_) and LRR20-CT (at B7) are predicted to be the most important interacting regions for PGN, LTA, and zymosan interactions, respectively. The structural organization of TIR domains in fish TLR2 and adapter molecule MyD88 have also been described. The interaction between TLR2-TIR and MyD88-TIR domain highlighted the contribution of BB loop, *α*B, *α*C, and CD loop in TLR2-TIR and BB loop, *α*B helix, and CD loop in MyD88-TIR domain. The data generated in this study are likely to be helpful to conduct further *in vivo *study to develop the strategy of innate immune activation and disease prevention in fish.

## Supplementary Material

Figure S1. Multiple sequence alignment of rohu TLR2 with other species by MegAlign program. N-termini, C-termini, LRR, trans-membrane (TM) and TIR domain regions are labeled. Conserved residues are shown inside yellow box. Majority axis represents the highest occurrence of a residue in a column.
Figure S2. Ramachandran plot analysis of (a) TLR2-ECD, (b) TLR2-TIR and (c) MyD88-TIR model. The plot was calculated in PROCHECK program.
Figure S3. DIMPLOT analysis of interaction between TLR2-TIR and MyD88-TIR domains (a) HADDOCK analysis and (b) ZDOCK analysis.
Figure S4. The DIMPLOT analysis of TLR2-TIR and MyD88-TIR complex after 10 ns of MDS.
Table S1. Cross-check validation report of homology models.
Table S2. List of predicted binding sites in rohu TLR2-ECD.
Table S3. The predicted interface residues in TLR2-TIR and MyD88-TIR domains.

## Figures and Tables

**Table 1:** Sequence identities between rohu TLR2-ECD (target) and mouse TLR2-ECD (template).

LRR	Identity (%)	LRR	Identity (%)
1	47.82	12	27.77
2	29.16	13	20
3	63.63	14	33.33
4	52	15	37.93
5	34.46	16	34.78
6	35.57	17	50
7	9.52	18	52.38
8	15.38	19	50
9	28.57	20	40.9
10	26.31	21	29.16
11	28		

**Table 2:** Sequence identities between rohu TLR2-TIR (target) and human TLR2-TIR (template).

Position	Identity (%)	Position	Identity (%)
*β*A	100	CD loop	46.66
AA loop	66.67	*β*D	60
*α*A	58.33	DD loop	69.23
*β*B	42.85	*α*D	88.88
BB Loop	100	DE loop	50
*α*B	90.9	*β*E	100
*β*C	75	EE loop	50
*α*C	69.23	*α*E	50

**Table 3:** Sequence identities between common carp MyD88-TIR (target) and human MyD88-TIR (template).

Position	Identity (%)	Position	Identity (%)
*β*A	100	CC loop	66.66
AA loop	80	*β*D	60
*α*A	69.23	DD loop	70.58
*β*B	100	*β*E	100
BB Loop	86.66	EE loop	66.66
*α*B	100	*α*E	68.75
*β*C	85.71		

**Table:** (a) Validation by SAVES server

Ramachandran plot (PROCHECK)	TLR2-ECD	TLR2-TIR	MyD88-TIR
	Residue (%)	Residue (%)	Residue (%)
Most favored regions	66.7	78.7	73.2
Additionally allowed regions	30.0	20.6	24.4
Generously allowed regions	1.8	0.0	1.6
Disallowed regions	1.6	0.7	0.8
Verify3D score	95.37	97.28	87.6
ERRAT	61.059	86.364	86.325
PROVE (mean *Z*-score)	1.609	1.48	1.63

**Table:** (b) Stereochemical quality of homology models by ProQ, ModFOLD, and MetaMQAP server

	TLR2-ECD	TLR2-TIR	MyD88-TIR
ProQ (LG/MX)	7.062/0.432	6.401/0.772	7.067/0.847
ModFOLD (Q/P)	0.6326/0.0065	0.7473/0.00038	0.5787/0.0022
MetaMQAP (GDT/RMSD)	68.93/2.137	78.767/1.523	72.628/2.319

*ProQ-LG: >1.5 fairly good; >2.5 very good; >4 extremely good. ProQ-MX: >0.1 fairly good; >0.5 very good; >0.8 extremely good. ModFOLD-Q: >0.5 medium confidence; >0.75 high confidence. ModFOLD-P: <0.05 medium confidence; <0.01 high confidence. MetaMQAP-GDT/RMSD: an ideal model has a GDT score over 59 and an RMSD around 2.0 Å.

**Table:** (a) Docking analysis of TLR2-ECD with PGN, LTA, and zymosan by AutoDock 4.0

Grid centre and ligand	Interacting residues	Binding energy kcal/mol	No. of H-bonds^a^
PGN-I (B5)	Tyr366, Thr395, Asn397, Ser399, Tyr421, Asn423, Ser425, His426, Asn446, Ser448, Ser449, Asp467, Glu470, Thr497	−4.29	6
PGN-I (B6)	Ser259, Ser285, Tyr286, His312, Thr313, Arg342, Ser344, Tyr366	−4.33	6
PGN-DAP (B5)	Asp368, Leu369, Ser370, Gln371, Asn397, Ser399, Gln400, Tyr421, Asp423, Ser425, His426, Asn446, Ser448, Ser449, Asp467, Ser469, Glu470, Thr489	−3.19	11
PGN-DAP (B6)	Thr246, Glu254, Gly255, Lys258, Leu276, Thr277, Met279, Asp280, Gly281, Ser282, Ser283, Leu284, Ser304, Tyr305, Thr306, His307, Tyr308, Glu309	−3.38	7
LTA sites	Glu323, Phe324, Phe325, Glu326, Met330, Met331, Phe335, Thr349, Val350, Phe351, Val352, Ile353, Pro354, Pro355, Ile356, Leu360, Asn372, Leu373, Leu374, Pro381	−1.92	1
Zymosan	Leu473, Thr474, Val475, Phe476, Asn477, Thr495, Leu496, Pro497, His498, Gly499, Glu500, Leu501, Ser520, Ser521, Asp522, Arg525	−7.55	13

^a^Hydrogen bonds.

**Table:** (b) Docking analysis of TLR2-ECD with PGN, LTA, and zymosan by FlexX 2.1

Ligands	Interacting residues	Binding energy kcal/mol	No. of H-Bonds^a^
PGN-I	Asn397, Ser399, Gln400, Asn401, His426, Ser428, Lys451, Glu470	−18.85	18
PGN-II	Ser428, Phe429, Val430, Ser448, Lys451, Arg453, Lys454, Asp472, Ser469, Gln470	−12.80	14
PGN-DAP	Gln400, Asn401, His426, Ser428, Ser449, Lys451	−15.47	15
LTA	Asn318, Leu319, Asp320, Ile321, Phe324, Asn347, Gly348, Thr349, Val350, Gln371	−6.92	13
Zymosan	Arg492, Leu493, Met494, Leu496, Arg516, Met517, Ser520, Asp522	−13.81	10

^a^Hydrogen bonds.

**Table:** (c) Docking analysis of TLR2-ECD with PGN, LTA, and zymosan by GOLD 4.1

Ligands	Interacting residues	GOLD fitness score	No. of H-Bonds^a^
PGN-I (B5)	Ile394, Asn397, Ser399, Gln400, Tyr421, Asp423, Ser425, His426, Asn446, Ser448, Ser449, Asp467, Ser469, Glu470, Thr489, Gly490, Glu511, Arg512	42.38	17
PGN-II (B5)	Tyr366, Asp368, Ser370, Gln371, Asn397, Ser399, Gln400, Tyr421, Ser425, Asn446, Ser448, Val465, Asp467, Ser469, Glu470, Thr489, Gly490, Arg512	44.01	20
PGN-DAP (B5)	Asn446, Ser448, Ser449, Asp467, Ser469, Glu470, Ile487, Thr489, Gly490, Gln511, Arg512	40.55	13
PGN-II (B6)	Thr246, Glu247, Pro248, Phe249, Lys250, Thr252, Thr277, Asp280, Ser304, Tyr305, Thr306, His307, Tyr308,	23.00	8
LTA	Leu319, Asp320, Ile321, Phe324, Phe327, Met330, Met331, Phe335, Gly348, Thr349, Val350, Phe351, Glu380, Pro381	44.65	4
Zymosan	Arg492, Leu493, Met494, Thr495, Leu496, Ala514, Leu515, Arg516, Met517, Phe518, Asn519, Ser520, Ser521, Asp522, Arg525	39.71	8

^a^Hydrogen bonds.

**Table 6:** List of hydrogen bond forming and hydrophobic interacting residues between TLR2-TIR and MyD88-TIR domains.

Hydrogen bonds			Hydrophobic interactions	
Donor	Acceptor	Length (Å)	Donor	Acceptor
Trp689-NE1	Leu84-O	1.97	Ala73	Gln654
Ser715-O	Ser50-N	2.34	Gln77	His655
Ser715-O	Ile48-O	2.84	Tyr8	Asp656
Ser715-OG	Asp36-OD2	2.78	Ser71	His680
Asp723-OD2	Lys55-NZ	2.70	Asp72	Lys681
Thr714-N	Asp36-OD2	2.62	Cys9	Arg682
Thr714-O	Asp36-O	2.72	Phe76	Phe684
Ser83-OG	Gly687-N	2.94	Lys79	Pro686
Gln654-NE2	Asp38-OD2	2.72	Leu82	Gly687
Ser11-SG	Asp656-OD1	3.15	Ser83	Trp689
Cys9-NZ	Asp656-OD1	2.70	Phe80	Ile690
Asp72-N	Asp656-OD2	2.56	Leu84	Glu710
Cys9-NZ	His655-O	2.61	Cys85	His711
Cys9-NZ	His655-ND1	2.78	Pro86	Val713
Pro686-O	Ser50-OG	2.14	Asp36	Thr714
Arg688-NH2	Glu51-OE2	2.75	Ala73	Gln654
Asp692-OD1	Lys10-NZ	2.20	Gln77	His655
Lys681-NZ	Asp72-OD2	2.73	Tyr8	Asp656
His655-NE2	Asp75-OD2	1.91	Ser71	His680
