# Supplementary material for: Elucidation of Novel Structural Scaffold in Rohu TLR2 and Its Binding Site Analysis with Peptidoglycan, Lipoteichoic Acid and Zymosan Ligands, and Downstream MyD88 Adaptor Protein
Source: Biomed Res Int. 2013 Jul 15;2013:185282. doi: 10.1155/2013/185282 (PMC3727187; doi:10.1155/2013/185282)
Supplement: Supplementary file 1 — Figure S1. Multiple sequence alignment of rohu TLR2 with other species by MegAlign program. N-termini, C-termini, LRR, trans-membrane (TM) and TIR domain regions are labeled. Conserved residues are shown inside yellow box. Majority axis represents the highest occurrence of a residue in a column. Figure S2. Ramachandran plot analysis of (a) TLR2-ECD, (b) TLR2-TIR and (c) MyD88-TIR model. The plot was calculated in PROCHECK program. Figure S3. DIMPLOT analysis of interaction between TLR2-TIR and MyD88-TIR domains (a) HADDOCK analysis and (b) ZDOCK analysis. Figure S4. The DIMPLOT analysis of TLR2-TIR and MyD88-TIR complex after 10 ns of MDS. Table S1. Cross-check validation report of homology models. Table S2. List of predicted binding sites in rohu TLR2-ECD. Table S3. The predicted interface residues in TLR2-TIR and MyD88-TIR domains. [file 185282.f1.pdf]

Table S1. Cross-check validation report of homology models

Cross-check	Ramachandran plot statistics by PROCHECK					
	2Z81		1O77		2Z5V	
	**X-ray**	**HM**[a]	**X-ray**	**HM**	**X-ray**	**HM**
Most favored regions	82.9	86.4	81.4	88.5	75.4	86.6
Additionally allowed regions	16.7	10.2	17.1	9.2	23.8	11.8
Generously allowed regions	0.4	2.6	1.6	0.8	0.8	0.8
Disallowed regions	0.0	0.8	0	1.5	0	0.8

[a] HM- homology models

Table S2. List of predicted binding sites in rohu TLR2-ECD

Binding sites	Amino acids
B1	Thr273, Leu276, Val278, Ser304, Leu311
B2	Lys236, Arg241, Glu247, Phe275, Val278,
B3	Lys168, Leu177, Phe182, Leu187, Ser194, Leu206, Leu223
B4	Ala534, Gly535, Asn537, Val540, Leu555, Val567
B5	Asn392, Leu396, Ser399, Tyr421, Asp423, Phe444, Ser448, Ser469
B6	Ser245, Thr248, Lys258, Leu272, Val278, Asp280
B7	Ile487, Thr495, Leu496, Met517, Asn519, Asp522
B_{mouse}	Asn318, Asp320, Gly323, Gln332, His341, Gly348, Val350

Table S3. The predicted interface residues in TLR2-TIR and MyD88-TIR domains

	TLR2-TIR	MyD88-TIR
**cons-PPISP**	H672, A676, C678, D683, F684, P686, G687, R688, W689, V691, D692, I694, D696, I698, S701, R719, L722, S725, H726, R728, H733, S736	C44, V45, A49, S50, E51, L52, E54, K55, R56, R59, K79, F80, L82, S83, L84, C85, P86, W127, R129, L130, K133, S136, L137
**PatchDock**	R645, Y646, V650, S651,Q685, I698, E699, K700, S701, H702, T704, L722, H726, F727, R728, I729, D731, H733, N734, D735	A81, L82, S83, L84, P86, G87, T88, H89, S90, K91, R92, L109, R110, F111, C115, D116, T118, R119, Q123, S121, F126
**InterProSurf**	D646, D647, I663, V665, P666, F675, A676, L677, I690, V691, D692, I694, S701, H702, R703, S725, H726, F727, R728, I729, V738, P751, L767, E768	C9, S11, H17, E18, K21, C33, F35, V39, W46, T47, F76, Q77, F80, L82, S83, H89, P104, F105, L109, R110, F111, I114, C115, D116, W125, F126, W127, I128, R129, A131, L135, S136

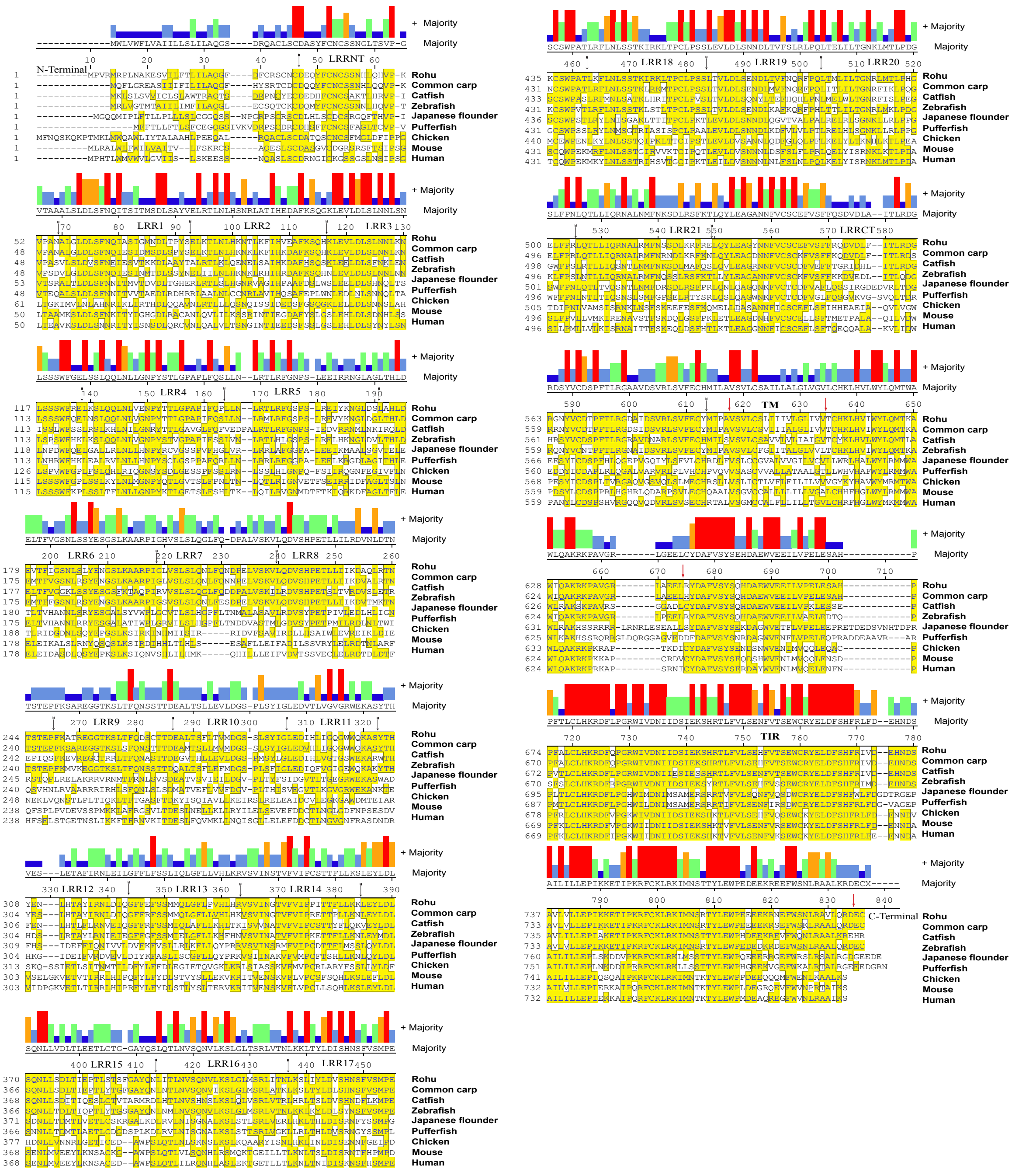

Supplementary figure 1. Multiple sequence alignment of rohu TLR2 with other species by MegAlign program

N-termini, C-termini, LRR, trans-membrane (TM) and TIR domain regions are labeled. Conserved residues are shown inside yellow box. Majority axis represents the highest occurrence of a residue in a column.

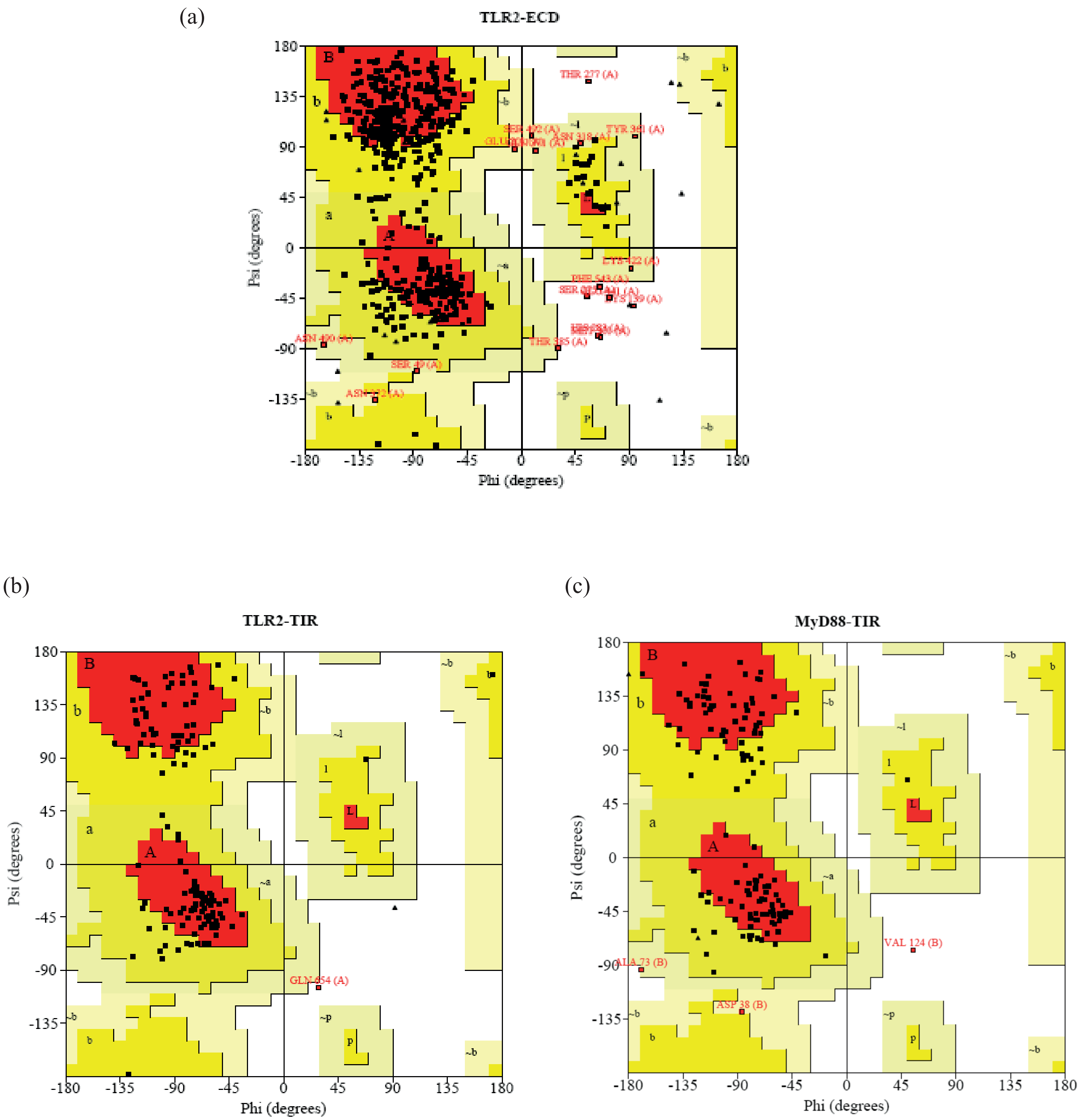

Supplementary Figure 2.
Ramachandran plot analysis of (a) TLR2-ECD, (b) TLR2-TIR and (c) MyD88-TIR model. The plot was calculated in PROCHECK program

(a)

(b)

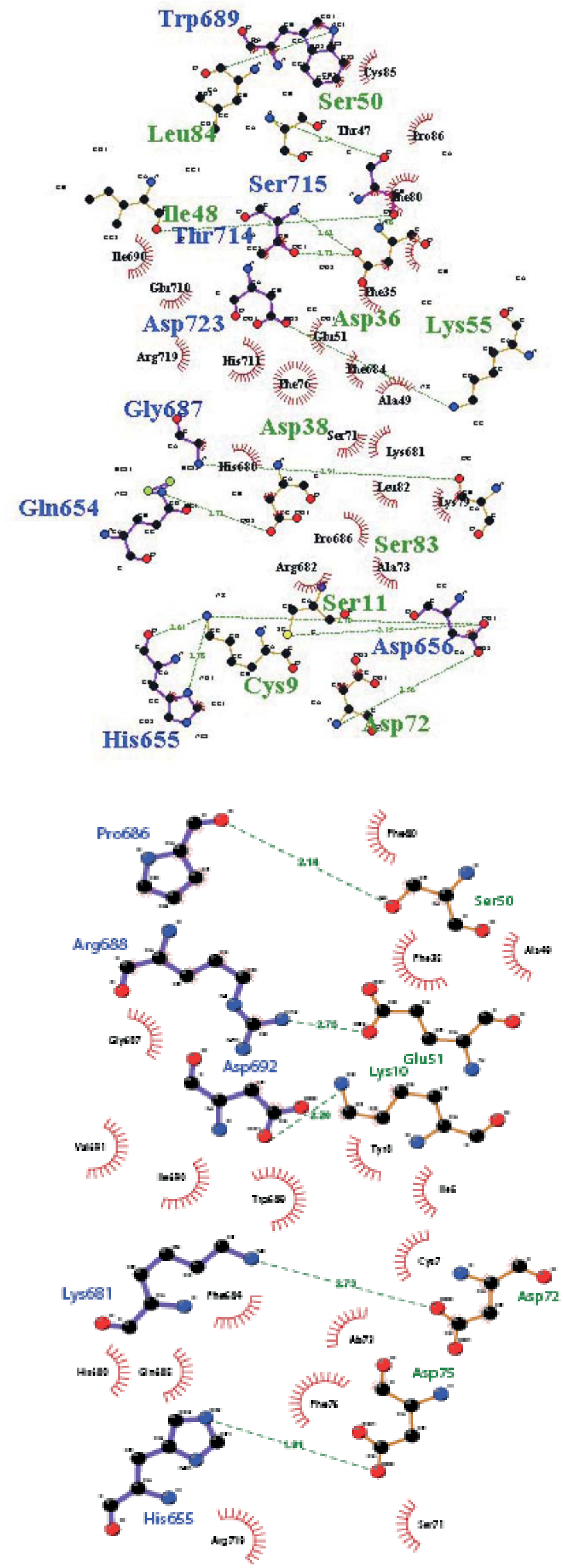

Supplementary Figure 3.
DIMPLOT analysis of interaction between TLR2-TIR and MyD88-TIR domains (a) HADDOCK analysis and (b) ZDOCK analysis

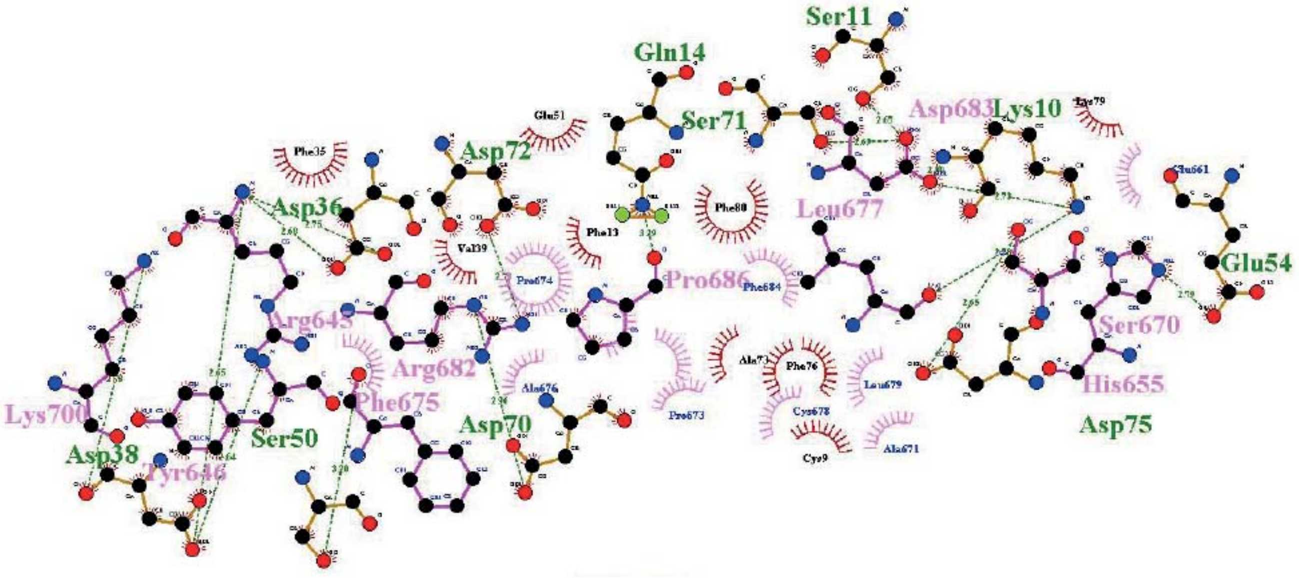

Supplementary Figure 4.

The DIMPLOT analysis of TLR2-TIR and MyD88-TIR complex after 10 ns of MDS
